# New Drugs, Appliances, and Things Medical

**Published:** 1892-01-30

**Authors:** 


### NEW DRUGS, APPLIANCES, AND THINGS MEDICAL.

[All preparations' appliances, novelties, etc., of which a notice is desired, should be sent for The Editor, to care of The Manager, 140, Strand, London, W.C.]

### SALIPYRIN.

Messrs. Allen and Hanburys have forwarded to us samples of the above. It is a crystalline compound of antipyrin and salicylic acid, and has a great vogue in Germany as a remedy for influenza. It is but slightly soluble in water, though readily so in alcohol. It melts at 91·5 deg., and contains 57·7 parts per 100 of antipyrin, and 42·3 of salicylic acid. It is stated to produce less sweating than the salicylates, and to have no ill effects on the heart. Certainly this latter property is extremely valuable, as we have again recently heard of several cases of antipyrin poisoning in persons recovering from the influenza. We have tried this remedy in several cases, with very fair results. It relieves the headache and the limb pains, but seems very like antipyrin in that if it does relieve soon further employment is practically non-indicated, as increasing the dose still leaves the condition unaltered. The dosage is rather large, a half dram to begin with, followed by four 15 grn. doses at intervals of an hour, seems to have produced very good results in a case of acute pneumonia under Dr. Sansom's care. It is also extremely useful in rheumatic fever, relieving the pain and reducing the temperature. If future experience should endorse, and we see no reason why it should not, the favourable opinion we have of this new combination drug, we think that chemical synthesis has again come powerfully to the aid of suffering humanity.

### MESSRS. CADBURY AND CO.'S COCOA AND CHOCOLATE PRODUCTIONS.

There are few to whom Messrs. Cadbury are not old and tried friends. We cannot for this reason, however, pass over the constant progress which they make towards perfection in their particular branch of trade. They have had to face formidable rivals, apart from their English competitors. When public taste revolted against the starchy mixtures sold under the name of cocoa, Van Houten's productions became deservedly popular. Messrs. Cadbury, however, have not been behind in the field, and their cocoa essence can compare favourably with any like productions offered to the public. In using this preparation in preference to any other the English public can be patriotic without any cost to themselves, nor are they likely after having given this cocoa a trial, to leave it in favour of any other. It is thoroughly pure, wholesome, and nutritious, and moderate in price. Whilst especially recommending this preparation, we must not forget to remind those who still remain faithful to the more antiquated forms of cocoa, whose principal characteristic is pasty thickness, that in deference to all requirements, this kind can also be procured from Messrs. Cadbury. Their chocolate compounds are equally comprehensive, and we congratulate them on enabling the British consumer to procure every variety of chocolate bonbon and fondant that can be procured over the water, and at a more moderate cost. Their chocolate ginger is especially excellent in our opinion, but where all tastes are provided for it is difficult to make a distinction.

### NEW VARIETY OF EUCALYPTUS OIL.

From Timbury's Eucalyptus Oil Co., 124, Clerkenwell Road, we have received a sample of their new variety of eucalyptus oil. It is a remarkably pure speciman, but what is most noticeable is that the odour is quite different from that of the oil of the Eucalyptus globulus and other varieties of the gum tree; this has a perfume very like oil of geranium, but with a distinct character of its own. It is distilled from the Eucalyptus maculata var. citriodora. As these volatile blue gum preparations are now in great request, we fancy many will prefer this variety to the others, as it is both pleasant and well adapted for the usual purposes of disinfection.

